# Implementation of exclusive enteral nutrition in pediatric Crohn's disease: a secondary analysis of the CLARA registry study

**DOI:** 10.3389/fped.2026.1787736

**Published:** 2026-05-26

**Authors:** L. Tischler, A. Boerkoel, S. M. Cantez, H. Krause, J. de Laffolie, N. van den Berg

**Affiliations:** 1 Institute for Community Medicine, University Medicine Greifswald, Greifswald, Germany; 2 German Center for Child and Adolescent Health (DZKJ), Partner Site Rostock/Greifswald, University Medicine Greifswald, Greifswald, Germany; 3 Department of General Pediatrics and Neonatology, University Children's Hospital, University Giessen, Giessen, Germany

**Keywords:** CEDATA, CLARA study, enteral nutrition, inflammatory bowel disease, Crohn's disease, pediatrics

## Abstract

**Background:**

Exclusive enteral nutrition (EEN) is the first-line therapy for children with active luminal Crohn's disease (CD). However, its implementation in routine care is still limited. Given the impact of the disease on age-appropriate development and quality of life in children and adolescents, as well as the rising incidence rates, the use of this effective and low-risk therapy should be intensified. Previous studies have demonstrated that patient registries can provide support in following guideline-based care. This study represents a secondary analysis of the CLARA cluster-randomized trial and aims to describe real-world patterns of enteral nutrition (EN) use and implementation gaps for children with luminal CD.

**Methods:**

This secondary analysis was based on data from a cluster-randomized, controlled trial (DRKS00015505). In the intervention group (IG), treatment providers documented the care of children and adolescents with inflammatory bowel disease (IBD) in the CEDATA-GPGE patient registry and received automated feedback on entered data and potential deviations from guideline recommendations (care deficits). In the control group (CG), treatments were documented as usual in patients' charts only. For this secondary analysis, initial treatment strategies for children with luminal CD during the first 90 days after diagnosis were compared between centers with registry-based documentation (IG) and those without (CG). The primary outcome was the documented use and pattern of EN, including the duration of EEN and combination therapies.

**Results:**

Of the 319 children recruited (IG: 21 centers, *n* = 160 patients; CG: 26 centers, *n* = 159 patients), 170 patients were diagnosed with CD (IG: *n* = 88; CG: *n* = 82). A total of 110 patients were included in the analysis (IG: *n* = 58; CG: *n* = 52). A non-significant trend towards higher overall use of EN was observed in the IG (72.4% vs. 61.5%; *p* = 0.225). EEN for the recommended duration was uncommon in both groups (IG: 9.5%; CG: 18.8%). Patients who did not receive nutrition therapy as initial treatment (IG: *n* = 16; CG: *n* = 20) were predominantly treated with a combination of immunosuppressants, aminosalicylates (5-ASA), biologicals, and/or steroids (IG: *n* = 13; CG: *n* = 6). Overall, the use of steroids within the first 90 days after diagnosis was more frequent in the CG compared to the IG (CG: *n* = 22; IG: *n* = 16, *p* < Chi^2^ 0.028).

**Conclusion:**

Registry documentation was associated with a non-significant increase in overall EN use but did not improve adherence to guideline-recommended EEN. The findings highlight a substantial gap between guideline recommendations and real-world implementation of EEN, underscoring the need to address structural, professional, and patient-related barriers.

## Introduction

Crohn's disease (CD) is the most common form of inflammatory bowel disease (IBD) and is characterized by symptoms such as abdominal pain, diarrhea, rectal bleeding, and fatigue. Particularly in young patients, the chronic nature of the disease, along with weight loss and malnutrition, can have serious consequences for growth, development, and quality of life (1, 2). Exclusive enteral nutrition therapy (EEN) is shown to be the optimal treatment option for children and adolescents with active luminal CD. In this therapy, patients are exclusively nourished with specially designed oral or nasogastric tube feeding over a period of six to eight weeks (3).

Numerous studies have shown that EEN achieves remission rates of 60%–80% (4–7), making it equivalent to or superior to other therapies (e.g., corticosteroid induction therapy). Moreover it can be conducted long-term without the use of biologics or surgical interventions (8, 9). The major advantage of minimizing steroid use lies in avoiding both short- and long-term side effects, such as poor growth, increased susceptibility to infections, and metabolic disturbances (Cushing's syndrome, osteoarthritis) (5, 10–13). The use of EEN for remission induction is recommended by many major professional organizations (ECCO, ESPGHAN, NASPGHAN, and ESPEN) and represents first-line therapy for children with luminal CD, regardless of disease severity (4, 8, 14).

Despite this, the use of EEN in initial treatment varies, with significantly more routine applications in Europe and Australasia compared to North America (14–16). The implementation of EEN seems to be influenced by how familiar pediatric gastroenterologists are with the use of EEN in their training and current clinical practice environment (17). Data from the German statutory health insurance provider AOK showed for 2014–2018 that only 18.1% of affected insured patients received EEN treatment (18). However, little is known about how frequently EEN is used as an initial therapy for children and adolescents with IBD in the clinical practice of specialized pediatric gastroenterology centers in Germany.

The CEDATA-GPGE registry, described in the scientific literature as one of the largest pediatric IBD (PIBD) registries based on the number of registered patients, enables pediatric gastroenterologists in Germany and Austria to systematically document the diagnosis, treatment, and disease course of PIBD (3, 18). Comparable large-scale initiatives include international registries such as PIBD-SETQuality (19), as well as European and national cohorts like EUROKIDS and population-based registries in North America (20, 21).

Within this framework, the cluster-randomized CLARA study aimed to improve the quality of care for PIBD patients by implementing registry-based algorithms and patient-specific feedback, highlighting deviations from guideline-recommended care. The primary goal was to reduce individual care deficits through structured feedback to healthcare providers (22).

Such registry-based feedback interventions can be conceptualized as a form of audit and feedback, a well-established strategy to improve professional practice. Evidence from a Cochrane review suggests that these interventions can result in small to moderate improvements in clinician behavior, particularly when deviations from guideline-based care are explicitly highlighted (23). This effect is thought to operate through self-regulation processes, whereby clinicians compare their current practice with defined standards and adjust their behavior accordingly (24). In contrast to formal clinical decision support systems, the CEDATA-GPGE registry does not provide direct treatment recommendations. However, its structured documentation framework and the integration of guideline-relevant information may facilitate guideline-concordant decision-making. By highlighting deviations from recommended care and providing patient-specific feedback, the registry may increase awareness of unmet care standards, such as the underuse of EEN, and thereby support adherence to evidence-based treatment strategies.

## Objective

As a secondary analysis of the CLARA study, we aimed to describe the real-world use, duration, and implementation patterns of EN therapies, with a particular focus on EEN, in children with luminal CD treated at registry vs. non-registry centers.

## Methodology

### Study design and participants

This was a controlled, cluster-randomized study (CLARA study) involving gastroenterology centers (clinics and practices) that had not yet registered their patients in the online CEDATA-GPGE registry at the start of the project. The centers included children with a confirmed diagnosis of IBD, whose date of diagnosis was no more than three months prior to the first presentation and who were under 18 years of age at that time. Data collection for the CLARA study took place between March 1, 2021, and May 31, 2022. For this purpose, one initial report form and two follow-up forms (after 6 and 12 months) had to be available for each patient. A time deviation of ±12 weeks was permitted.

### Intervention

In the intervention group (IG), healthcare providers documented the diagnostic and therapeutic details of their patients in the CEDATA-GPGE patient registry via the online platform. There was no feedback from the registry regarding the EEN therapy parameter. In the control group (CG), providers did not document in the registry during the observation period. After the observation period, the diagnostic and treatment data from the CG centers were retrospectively retrieved from the patients' medical records and transferred into the CEDATA-GPGE registry by the study team.

### Endpoint

The primary endpoint of this secondary analysis was the documentation and pattern of EN use, including:

- Any EN use,
- EEN,
- duration of EN/EEN,

and combination with pharmacological therapies.

### Data analysis

For this analysis, children with luminal CD were included if they did not have prognostic risk factors such as extra-intestinal manifestations or perianal disease (25). These specific complications require particular therapeutic approaches that cannot be addressed solely by EEN.

For the included patients, the data on initial therapy (the first three months of treatment after diagnosis) were reviewed in the initial report forms and categorized according to pre-defined groupings established by a pediatric gastroenterologist (see Table 1). The therapy categories included immunomodulators (mercaptopurine, azathioprine), 5-ASA (5-aminosalicylic acid, mesalazine, and sulfasalazine), biologicals (infliximab, adalimumab), and steroids (budesonide, prednisone, dexamethasone, methylprednisolone).

**Table 1:** Treatment of children with luminal Crohńs disease with and without nutritional therapy in both study groups.

Treatment with EN	IG (*n* = 42)		CG (*n* = 32)	
**Type of therapy (grouped)**	***n***	**%**	***n***	**%**
EEN	4	9.5	6	18.8
EN and immunomodulators	21	50.0	5	15.6
EN and combination of immunomodulators, 5-Asa, biologicals, steroids	15	35.7	18	56.3
EN and operation	0	0.0	1	3.1
EN and other	2	4.8	2	6.3

**Treatment without EN**	**IG (*n*** **=** **16)**		**CG (*n*** **=** **20)**	
**Type of therapy (grouped)**	***n***	**%**	***n***	**%**
Immunosuppression	1	6.3	1	5.0
5-ASA	1	6.3	3	15.0
Biologicals	0	0.0	2	10.0
Steroids	1	6.3	4	20.0
Combinations of immunomodulators, 5-Asa, biologicals, steroids	13	81.3	6	30.0
Operation	0	0.0	0	0.0
Not included in ECCO/ESPGHAN guidelines	0	0.0	2	10.0
No specification/documentation	0	0.0	2	10.0

EN, enteral nutrition; EEN, exclusive enteral nutrition; IG, intervention group; CG, control group; 5-Asa, 5-Aminosalicylic acid; ECCO, European Crohn’s and Colitis Organization; ESPGHAN, European Society for Pediatric Gastroenterology Hepatology and Nutrition.

Descriptive statistics were calculated for the included centers and patients. The frequency of prescribed EN therapy and steroid use within the first three months of treatment was compared between groups using chi-square tests (*α* = 0.05). A *post-hoc* power analysis was conducted based on the observed proportions of EN use in the intervention group and control group with a significance level of *α* = 0.05.

According to current clinical guidelines, EEN for induction therapy in children with luminal CD is recommended for approximately 6–8 weeks (5). In the registry used for this study, therapies administered during the initial treatment phase were documented separately for the first three months after diagnosis (month 1, month 2, and month 3). To operationalize adherence to the recommended treatment duration, guideline-concordant EEN therapy was defined as documentation of EN in at least two of these three months, reflecting a treatment duration of approximately six weeks or longer in routine clinical practice.

To assess potential factors influencing the use of EN and to account for baseline differences between participating centers, a multivariable logistic regression analysis was performed. The dependent variable was the use of EN as part of the initial therapy. Independent variables included study group (intervention vs. control), center size (<50 vs. ≥50 patients with PIBD treated per year), and GPGE certification status (yes/no). Odds ratios with corresponding likelihood ratio tests were calculated to assess the independent association of these variables with EN use.

Descriptive and univariate analyses were performed using IBM SPSS Statistics for Windows, Version 29.0.1.1 (IBM Corp., Armonk, NY, USA), while additional regression analyses were conducted using appropriate statistical software. All analyses followed the principles of Good Epidemiological Practice (26) and the CONSORT guidelines for cluster-randomized trials (27).

## Results

### Participants

**Figure 1:**
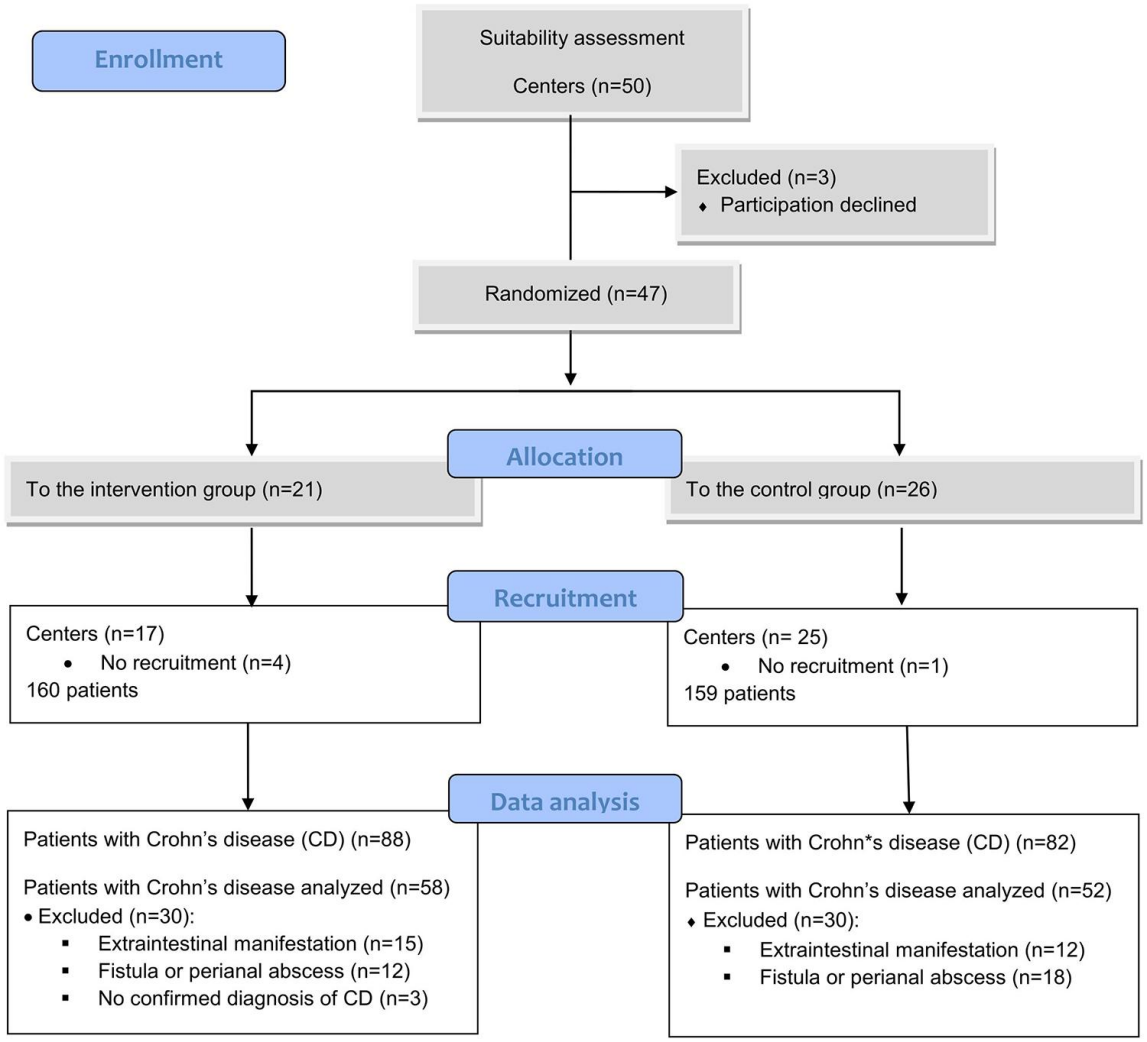
Consort flow diagram.

A total of 47 pediatric gastroenterology centers in Germany participated in the CLARA study (IG = 21 centers; CG = 26 centers), recruiting 319 patients in total (IG = 160; CG = 159). Of these, 170 patients (IG = 88; CG = 82) were diagnosed with CD. After applying the exclusion criteria, 58 of the 88 children in the IG and 52 of the 82 patients in the CG were included in the analysis (see Figure 1).

Table 2 provides an overview of the sample characteristics. In both groups, a higher proportion of male patients with CD was observed. The proportion of female patients was slightly higher in the CG than in the IG (IG: 36.2%; CG: 48.1%). In the CG, a higher proportion of centers (95.7%) treated fewer than 50 children with PIBD in 2019 compared to the IG (59.6%). The median age of patients in both groups was 14 years.

**Table 2:** Sample characteristics of the study groups.

Sample characteristics		IG (*n* = 58)		CG (*n* = 52)	
Sex	Female (*n*;%)	21	36.2	25	48.1
Age	Median (SD)	14 (2.88)		14 (3.52)	
Sector	Hospital (*n*;%)	57	98.3	46	88.5
	Out-patient (*n*;%)	1	1.7	6	11.5
Number of patients with PIBD/center^a^	<50 patients/year	34	59.6	44	95.7
	50–100 patients/year	14	24.6	2	4.3
	<100 patients/year	9	15.8	0	0.0
GPGE-certification	Yes	32	55.2	36	69.2

a Hospital quality reports from the 2019 reporting year.

IG, interventions group; CG, control group; PIBD, Pediatric Inflammatory Bowel Disease; GPGE, German Society for Pediatric Gastroenterology and Nutrition; SD, standard deviation.

### Endpoints

#### Use of EEN and EN

Table 1 shows that 72% (*n* = 42) of patients with CD in the IG, whose treatment was documented in the CEDATA-GPGE registry, and 61.5% (*n* = 32) of patients with CD in the CG received EN within the first three months (*χ*^2^-test, *p* = 0.225). EEN was documented in 9.5% of patients in the IG and 18.8% of patients in the CG.

The estimated statistical power to detect the observed difference of 10.9 percentage points between groups (IG 42/58, 72.4%; CG: 32/52, 61.5%) was approximately 23%, indicating limited power due to the modest sample size.

Using this operational definition, documentation of EN in at least two of the first three months was observed in 36 of 42 patients (84.7%) in the IG and in 16 of 32 patients (50.0%) in the CG. More specifically, in the IG, 26 of 42 patients (61.9%) received EN continuously over all three months, whereas in the remaining patients, EN was documented for a shorter duration. In four cases, early discontinuation was documented, including three due to non-compliance and one due to intolerance. In the CG, EN was applied for the entire first three months in 10 of 32 patients (31.3%).

In the IG for 23 patients, information was documented about the duration of EN therapy. The median duration was 50 days (IQR 37.55–63.04). In the CG, for 6 patients, information was documented about duration, with an average of 63 days (IQR 44.58–86.80).

#### Combination with pharmacological therapies

In the IG, half of the patients received EN in combination with immunomodulators (e.g., methotrexate, azathioprine) within the first three months. Most patients in the CG (56.3%) received a combination of several medications alongside EN (e.g., EN + immunomodulators + corticosteroids/antibiotics/biologicals).

Among patients who did not receive EN in the IG, 81.3% were initially treated with combination therapy (e.g., immunomodulators and corticosteroids). In the CG, this was true for 30,0% of patients. Additionally, 20.0% of patients with CD in the CG were initially treated with steroids. Furthermore, 2 patients (10.0%) in the CG received a therapy not included in the guidelines.

Table 3 shows that 44.3% of children with luminal CD in the CG received steroids in their initial treatment, with 7.7% of patients treated with steroids only. In the IG, the use of steroids for remission induction was less frequent (29.3%). Steroids were used significantly more frequently in the CG than in the IG (*p* < Chi^2^ 0.028).

**Table 3:** Provided treatment of children with luminal Crohńs disease with and without steroids in both study groups.

Initial treatment with/without steroids	IG (*N* = 58)		CG (*N* = 52)	
**Type of therapy (grouped)**	**n**	**%**	**n**	**%**
EN and steroids	7	12.1	12	23.1
Steroids and other combinations	8	15.5	7	13.5
Only steroids	1	1.7	4	7.7
No treatment with steroids	42	70.7	27	51.9
No documentation	0	0.0	2	3.8

IG, intervention group; CG, control group; EN, enteral nutrition.

#### Center-level factors associated with EN use

A multivariable logistic regression analysis was conducted to adjust for potential center-level confounders (see Table 4). Center size (<50 vs. ≥50 patients with PIBD per year) and GPGE certification status were significantly associated with the use of EN. Larger centers were more likely to use EN as part of the initial therapy (*p* = 0.0013), and GPGE-certified centers also showed a significantly higher likelihood of EN implementation (*p* = 0.0034). In contrast, study group allocation (intervention vs. control) was not associated with EN use after adjustment for these variables (*p* = 0.85). Odds ratios with corresponding likelihood ratio tests were calculated to assess the independent association of these variables with EN use.

**Table 4:** Multivariable logistic regression analysis of factors associated with use of enteral nutrition.

Variable	OR	95% CI	*p*-value
Study group (IG vs CG)	1.04	[0.68–1.60]	0.85
Center size^a^ (≥50 vs <50 patients with PIBD per year)	2.80	[1.50–5.20]	0.001
GPGE certification (yes vs no)	2.08	[1.28–3.37]	0.003

a Hospital quality reports from the 2019 reporting year.

IG, intervention group; CG, control group; PIBD, Pediatric Inflammatory Bowel Disease; GPGE, German Society for Gastroenterology and Nutrition.

## Discussion

This secondary analysis of the CLARA study examined real-world patterns of EN use in children with luminal CD and focused on the implementation of EEN as a guideline-recommended first-line therapy. Overall, a higher proportion of patients in centers documenting care within the CEDATA-GPGE registry received some form of EN compared to patients treated by clinics not documenting in the registry. However, the relatively small sample size may have limited the ability to detect statistically significant differences between groups. The results do not demonstrate a statistically significant effect of registry documentation on adherence to EEN as first-line therapy. Instead, the data reveal a marked discrepancy between guideline recommendations and real-world clinical practice. While awareness of nutritional therapy appears high, sustained implementation of true EEN remains rare.

Although a higher proportion of patients in the IG received EN overall, EEN for the recommended duration was documented less frequently, suggesting that EN was predominantly used in combination with other therapies rather than as an exclusive induction strategy. A more detailed inspection of combination therapies revealed that EN was most commonly used in combination with steroids and immunomodulators, as well as with biologic therapies. At the same time, a broad spectrum of additional, less frequent combinations was observed, resulting in substantial heterogeneity and very small subgroup sizes. This limits the feasibility and interpretability of a more granular analysis of treatment patterns.

One possible explanation for the more frequent documentation of some form of EN among registry users may lie in the engagement with and data entry into the registry platform, which incorporates the most current treatment guidelines (28). Additionally, study participants (including the CG) may have been subject to an observation effect, meaning that, in the absence of such observation, even lower EN prescription rates may be expected.

A previous analysis of registry data for patients with PIBD from the CEDATA-GPGE registry (2004–2014) found that approximately 32% of children with CD received guideline-adherent treatment with EN as initial therapy (3). Between 2014 and 2018, this number increased to 51% (28). In comparison with our results, where 72% of patients received EN, the findings suggest an improvement in the implementation of first-line therapy through the standardization of documentation over time. However, only a few patients in our analysis received EEN for the entire 90-day period. For many patients, EN was used supplementarily. While EEN is considered a safe treatment modality, challenges remain regarding patient acceptance and long-term adherence (8, 9, 29).

In 2021, a prospective survey of CEDATA-GPGE clinics (members of the GPGE) found that almost 90% of children with CD were recommended EEN to induce remission, with 72% reportedly beginning therapy (30). International studies have shown a wide variation in the use of dietary interventions for CD, with rates ranging from 12% to 89% (31–33).

The long-term use of EEN, which involves a liquid-only diet with a taste that some patients may find difficult, can be a significant burden for both patients and their families. In addition to the strict adherence to dietary guidelines, some patients report gastrointestinal symptoms such as bloating or nausea at the beginning of therapy. Developing strategies to improve acceptability, such as offering taste variants or gradually reintroducing solid foods after a certain period, could be helpful. Modified approaches such as the Crohn's Disease Exclusion Diet (CDED), the Tasty and Healthy Diet and Partial Enteral Nutrition (PEN) are increasingly being used, with early studies suggesting that combining PEN with a specific diet may yield comparable outcomes (34). Recent recommendations further support the use of CDED in combination with PEN as a promising alternative to EEN, particularly with regard to improved tolerability and adherence (35). At the time of the CLARA study, however, these modified approaches were not yet considered equivalent to EEN.

Our findings suggest that registry documentation may help increase awareness of guideline-recommended first-line therapies, but is not sufficient on its own to ensure adherence to EEN in routine clinical practice. In addition, treatment patterns varied across centers, indicating that factors beyond registry participation play a relevant role. In line with the multivariable analysis, structural characteristics such as center size and GPGE certification appear to influence the implementation of EN. Larger and certified centers may benefit from greater clinical experience, established multidisciplinary infrastructures (e.g., dietitians, IBD nurses, psychologists), and more structured care pathways, which may facilitate the use of EEN in routine practice. Prescription patterns nevertheless varied across centers, suggesting that factors beyond registry also contribute. In addition to patient's adherence, physician's qualification and experience, availability of specialized personnel, and sufficient time for patient education appear to be key determinants for successful EEN implementation.

Identifying barriers to effective EEN treatment, such as tolerance issues, non-compliance, or psychological challenges, and subsequently providing support through a multidisciplinary team (MDT) approach (e.g., dietetics, specialized nursing, and psychological support) is crucial for improving patient adherence (18). Our data do not provide insight into why healthcare providers may choose to prescribe or withhold EEN in specific cases.

For children and adolescents with moderate to severe CD who do not respond to EEN within one to two weeks or those who refuse the dietary intervention, oral corticosteroid therapy is recommended for remission induction (36). This may explain why patients in the control centers were more frequently treated with steroids. While some studies have reported a superiority of EEN over corticosteroid therapy for the induction of remission in children with luminal CD (9, 37, 38), particularly with respect to sustained outcomes and mucosal healing (39–41), several meta-analyses have found no significant difference in overall remission rates between EEN and corticosteroids in pediatric populations (42–44). In contrast, corticosteroid therapy is associated with important limitations in children. Beyond the lack of consistent promotion of mucosal healing, corticosteroids carry a substantial risk of adverse effects, including growth retardation, reduced bone mineral density, and metabolic complications. Moreover, corticosteroid treatment is associated with steroid dependence in approximately 14%–50% of patients within one year (45). Based on an overall assessment of efficacy, safety, growth effects, and long-term prognosis, nutritional therapy should be preferred over steroid therapy (5, 46, 47).

### Limitation

This analysis has limitations due to its secondary nature and was not powered to detect small between-group differences. The imbalance in center size between study groups and retrospective data collection in the CG introduced potential confounders and information bias, which may limit the validity of direct group comparisons.

When comparing the groups, larger centers were more frequently represented in the IG. This may introduces a size-related bias, as larger centers typically have greater clinical experience and access to specialized resources, potentially influencing the observed outcomes.

Due to the retrospective data extraction in the CG, incomplete documentation in medical records may have resulted in missing information for certain variables, particularly the duration of EN therapy. However, the primary outcome (use of EN as part of initial therapy) was not affected by missing data, as treatment modalities were recorded as categorical variables within the registry.

The CLARA project took place during the COVID-19 pandemic, which may have impacted the data collection process. Due to the restrictions and limitations imposed by the pandemic, the study team was unable to conduct on-site data collection in the control centers. As a result, the centers in the CG were asked to send their complete patient documentation to the study center, where the data was extracted and entered into the registry. This change primarily affected the logistics of data acquisition, as direct on-site verification and clarification of data were not possible.

Data completeness was generally lower in the control group due to retrospective data extraction from medical records. However, no relevant differences in documentation completeness were observed within the CG before and during the COVID-19 pandemic, suggesting that these differences were primarily related to the mode of data collection rather than pandemic-related effects.

Retrospective data extraction outside of a structured registry setting may increase the risk of incomplete or inconsistent documentation (48), particularly in complex, cross-sector and interprofessional care settings such as PIBD (49). This may have affected the availability of clinical information, especially regarding inflammatory activity, and should be considered when interpreting treatment decisions in the CG.

Incomplete documentation of inflammatory activity limited the ability to fully assess disease severity and its influence on treatment decisions in the CG. As disease severity may influence therapeutic decision-making, this limits the interpretation of treatment choices in the CG. In addition, long-term outcome data were not available for analysis. Nevertheless, current ESPGHAN/ECCO guidelines recommend EEN as first-line induction therapy for children with luminal CD irrespective of disease severity, underscoring that EEN should be considered across the full spectrum of disease activity.

## Conclusion

The present analysis demonstrates that, despite widespread guideline recommendations, EEN remains poorly implemented in routine children with luminal CD care. Registry documentation may increase awareness of nutritional therapy but does not appear sufficient to ensure adherence to guidelines for EEN. Importantly, our findings suggest that structural characteristics of treatment centers—such as center size and specialized certification—play a key role in the implementation of nutritional therapy. Optimizing these structural conditions may therefore be as important as guideline dissemination in improving adherence to EEN. Future strategies should focus on targeted implementation support, strengthening multidisciplinary care models, and the evaluation of alternative dietary approaches to enhance both feasibility and acceptance in clinical practice.

## Data Availability

The raw data supporting the conclusions of this article will be made available by the authors, without undue reservation.
