# Supplementary material for: Implementation of exclusive enteral nutrition in pediatric Crohn's disease: a secondary analysis of the CLARA registry study
Source: Front Pediatr. 2026 May 26;14:1787736. doi: 10.3389/fped.2026.1787736 (PMC13246686; doi:10.3389/fped.2026.1787736)
Supplement: Supplementary file 1 [file Table1.docx]

**Clara study group**

| **centre** | **participating doctor** |
| --- | --- |
| Universitätsklinikum Aachen | Prof. Dr. Tobias Wenzel |
| Josefinum Augsburg | Dr. Christoph Schick,  Dr. Markus Richter |
| Universitätsklinikum Augsburg | Dr. Lisa Thammer |
| Kinderarztpraxis Willenborg Berlin;  St. Joseph Berlin | Dr. Ahlke Willenborg |
| Charité – Universitätsmedizin Berlin | Prof. Philip Bufler |
| Zentrum für Kinderheilkunde der Universität Bonn | Dr. Gesche Düker |
| Carl-Thiem Klinikum Cottbus | Dr. Simone Stolz |
| Helios Klinikum Duisburg | Dr. Rüdiger Kardoff |
| Universitätsklinikum Düsseldorf | Dr. Michael Friedt |
| Universitätsklinikum Erlangen | Dr. André Hörning |
| Universitätsklinikum Essen | Dr. Denisa Pilic |
| Klinikum Esslingen | Dr. Olaf Raecke |
| Kinderarztpraxis Forchheim | Dr. Thorsten Fröhlich |
| Universitätsklinikum Freiburg | Dr. Ulrike Teufel-Schäfer |
| Kinderklinik für Kinder und Jugendliche Friedrichshafen | Dr. Simone Jedwilayties |
| Klinikum Fürth | Dr. Tobias Rechenauer |
| Kinderarztpraxis Gründau | Dr. Stefan Sgoll |
| Katholisches Kinderkrankenhaus Hamburg | Dr. Florian Schmidt |
| Universitätsklinikum Hamburg-Eppendorf | Dr. Daniel Tegtmeyer |
| AKK Altonaer Kinderkrankenhaus Hamburg | Dr. Andreas Rieger |
| Praxis für Kinder- und Jugendgastroenterologie Hamburg | Dr. Thomas Schneider |
| Asklepios Klinik Hamburg | Dr. Daniela Nolkemper |
| Evangelisches Krankenhaus Hamm | Prof. Dr. Wolfgang Kamin |
| Kinderarztpraxis Hannover | Dr. Marc Bohn |
| Universitätsklinikum Jena | Dr. Steffen Reinsch |
| Klinikum Kassel | Prof. Dr. Andreas Jenke |
| Helios Klinikum Krefeld | Dr. Antje Ballauff |
| Kinderarztpraxis Künzell | Dr. Benedikt Pircher |
| Universitätsklinikum Leipzig | Dr. Gunter Flemming |
| Klinikum St. Georg Leipzig | Dr. Marlen Zurek |
| Universitätsklinikum Schleswig-Holstein  Campus Lübeck | Dr. Martina Kohl-Sobania |
| Klinikum Magdeburg | Dr. Matthias Heiduk |
| Universitätsklinikum Mannheim | Dr. Rüdiger Adam |
| Helios Klinikum Meiningen | Dr. Christoph Ehrsam |
| Klinikum Memmingen | Dr. Ralf Pallacks |
| Kinderklinik und Poliklinik München | Prof. Dr. Susanne Liptay |
| Universitätsklinikum Münster | Dr. Thomas Kaiser |
| Kinderarztpraxis Neu-Isenburg | Dr. Adrian Lieb |
| Klinikum Nürnberg Süd | Dr. Jochen Röhm |
| St. Vincenz Krankenhaus Paderborn | Dr. Viola Schulze |
| Harzklinikum Quedlinburg | Dr. Peter Klipstein |
| Helios Kliniken Schwerin | Dr. Esther Schmidt |
| Zentralklinikum Suhl | Dr. Christoph Ehrsam |
| Universitätsklinikum Tübingen | Dr. Ekkehard Sturm |
| Klinikum Worms | Dr. Andrea Arnoldy |
| Helios Universitätsklinikum Wuppertal | Prof. Dr. Stefan Wirth |
| Universitätsklinikum Würzburg | Dr. Anke Dick |

**CEDATA AG**.

| **member** | **institute** |
| --- | --- |
| Prof. Dr. Jan de Laffolie (1. Sprecher) | Justus-Liebig-Universität Gießen |
| Prof. Dr. Jan Däbritz (2. Sprecher) | Universitätsmedizin Greifswald |
| Prof. Dr. Philip Bufler | Charité – Universitätsmedizin Berlin |
| PD Dr. Stephan Buderus | St. Marien-Hospital Bonn |
| Dr. Sönke Dammann | Olgahospital Stuttgart |
| Dr. Michael Friedt | Universitätsklinik Düsseldorf |
| Dr. Andreas Krahl | Sana Klinik Offenbach |
| Dr. Angeliki Pappas | Universitätsklinik Aachen |
| Dr. Thomas Lang | Klinik St. Hedwig Regensburg |
| Prof. Dr. Carsten Posovszky | Universitätskinderkliniken Ulm, Zürich |
| PD Dr. Tobias Schwerdt | LMU München |
| Dr. Martin Laass | Universitätskinderklinik Dresden |
| Prof. Dr. Almuth Hauer | Medizinische Universität Graz |
| Dr. Stefan Trenkel | Kinderklinik Potsdam |
| Dr. Martin Claßen | Kinderklinik Bremen |
